# Bilateral Tubal Ectopic Pregnancy: A Case Report and Review

**DOI:** 10.7759/cureus.68742

**Published:** 2024-09-05

**Authors:** Janelle R Trefsgar, William Padgett

**Affiliations:** 1 Obstetrics and Gynecology, A.T. Still University, Mesa, USA; 2 Obstetrics and Gynecology, Southern Illinois Healthcare Foundation (SIHF) Healthcare, Olney, USA

**Keywords:** beta-human chorionic gonadotropin (β-hcg), bilateral ectopic pregnancy, bilateral salpingectomy, laparoscopic salpingostomy, methotrexate in ectopic

## Abstract

A bilateral ectopic pregnancy is a rare condition, and even more so with spontaneous conception. The known risk factors and clinical presentation are shared by both unilateral and bilateral ectopic pregnancy. This poses a risk for misdiagnosis, treatment failure, and, ultimately, maternal mortality. The current standard for diagnostics is not discernible for a bilateral ectopic pregnancy, thus, medical management tends to be sub-therapeutic. In fact, it is fairly common for the correct diagnosis and efficient treatment to be achieved by surgical intervention. As there are no established diagnostic or treatment guidelines for this rare condition, the possibility of a bilateral ectopic pregnancy should not be ruled out lightly.

## Introduction

An ectopic pregnancy (EP) is the implantation of a gestational sac in any site other than the uterus and occurs in 1.5-2% of all pregnancies [1]. A bilateral EP is defined as two gestational sacs implanted outside the uterus in areas opposite of one another. Ectopic implantation sites include the cervix, uterine cornea, fallopian tubes, ovaries, and abdominal cavity [1-3]. In more than 90% of cases, an EP is found within the fallopian tubes, specifically within the ampulla region. Bilateral tubal ectopic pregnancy (BTP) is the rarest form of pregnancy, with an incidence of 1 in 200,000 pregnancies [1]. BTPs can be spontaneous, meaning there is no involvement of assisted reproductive therapy (ART). Overall, 1 in 725 to 1,580 BTPs are spontaneous in origin, with the first spontaneous BTP published by Bledsoe in 1918 [1,4]. The known risk factors of BTP are no different than unilateral EPs, which include the use of ART, ovulation induction, intrauterine contraceptive devices (IUDs), sexually transmitted infections (STIs), pelvic inflammatory infections (PIDs), history of EPs, and tubal surgery [1,2]. However, about half of the patients diagnosed with EP have no known risk factors [2]. This report presents a case of spontaneous BTP without known risk factors that was diagnosed and treated with laparoscopic intervention.

## Case presentation

A 31-year-old female, G6P4014 (gravida 6, full term 4, preterm 0, abortion 1, living 4), presented to our emergency department (ED) with left flank pain for two days. Her last menstrual period was six weeks prior and she had a positive at-home pregnancy test before arriving at the ED. Obstetric history revealed four spontaneous, full-term vaginal deliveries and one spontaneous abortion treated with dilation and curettage six years ago. She had no known history of ART or STIs. Other pertinent medical history included a cholecystectomy, frequent urinary tract infections (UTIs), and daily cigarette and marijuana use. Vital signs were stable and the physical examination was positive for left costovertebral tenderness. Laboratory investigations showed a serum beta-human chorionic gonadotropin (β-hCG) of 3,434 mIU/mL, hemoglobin of 11.7 g/dL, hematocrit of 35%, and white blood cells of 13.02 µL. A urinalysis was consistent with a UTI with trace protein, small blood, positive nitrites, positive leukocyte esterase, and moderate bacteria. Her urine was sent for culture. The patient was administered 1,000 mL 0.9% NaCl infusion and 1 g ceftriaxone intravenously to treat the UTI. She was discharged same-day with 500 mg cephalexin, one capsule taken by mouth four times daily for seven days, and recommended to follow up with obstetrics and gynecology (OBGYN) within two days.

The patient called our clinic 12 days later to schedule an obstetrical ultrasound (US) and a new obstetric visit. There was no mention of pain at this time. A transvaginal ultrasound (Canon Aplio i800 ultrasound machine and a multi-frequency transducer (11C3)) was completed one week later, on the morning of her first obstetric visit. Ultrasound identified a 62 mm multinodular left adnexal mass suspicious for EP, a 34 mm heterogeneous right adnexal mass, a trace amount of free fluid present in both adnexae, and no fetus or products of conception identified.

After extensive discussion, the patient chose to pursue methotrexate (MTX) therapy over diagnostic laparoscopy. For this, 1.9 mL intramuscular MTX sodium was injected into both the right and left ventrogluteal sites. She was advised to seek medical care if she developed severe abdominal pain or heavy vaginal bleeding. In comparison to her ED labs, the only notable differences were a serum β-hCG of 18,001 mIU/mL, hemoglobin of 10.7 g/dL, hematocrit of 31.4%, and white blood cells of 6.98 µL.

Three days later, the patient called the clinic in excruciating pain and was advised to go to the ED, where the gynecologist would arrange to meet her. She presented to the ED for severe left lower quadrant pain with vaginal bleeding, weakness, and nausea. Vital signs were stable. Laboratory investigations showed a hemoglobin of 10.9 g/dL, hematocrit of 32.2%, and white blood cells of 7.8 µL. Physical examination of the abdomen was soft, but tender in both lower quadrants with rebound tenderness. Bedside transabdominal ultrasound revealed fluid in the cul-de-sac. Assessment at that time was a probable EP with signs and symptoms consistent with rupture. The patient agreed to and provided consent for a diagnostic laparoscopy with possible salpingectomy and possible dilation and curettage of the uterus.

**Figure 1:**
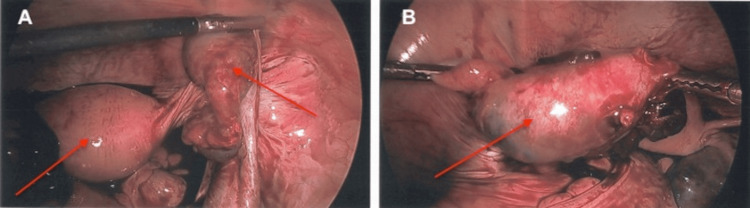
Right fallopian tube. (A) Laparoscopic view of the right fallopian tube (right red arrow) with respect to the uterus (left red arrow). (B) The right fallopian tube with mid-segment swelling and dilatation consistent with ectopic pregnancy.

**Figure 2:**
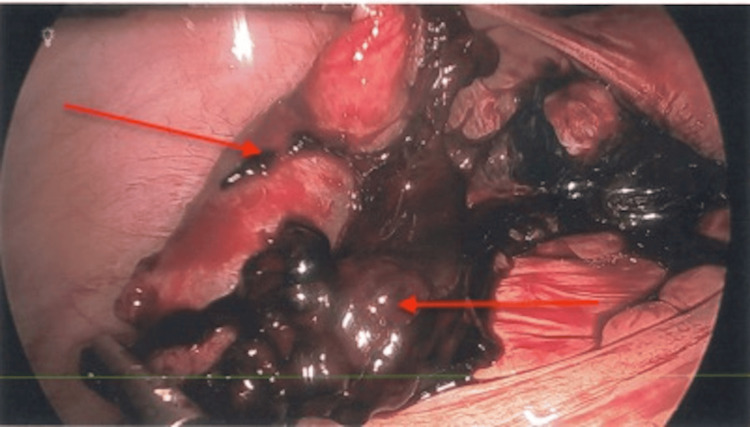
Left fallopian tube. Laparoscopic view of the edematous left fallopian tube (left red arrow) and blood clots (right red arrow).

Laparoscopic exploration identified approximately 100-150 mL of blood in the pelvis. The right fallopian tube had mid-segment swelling and dilatation consistent with EP, as shown in Figure 1. The right ovary and fimbria were seen below this and appeared to be normal. The left distal tube and ovary were adhesed to the pelvic sidewall and the sigmoid colon. The distal half of the left fallopian tube was edematous and bleeding, as shown in Figure 2, and consistent with EP. The left fimbria could not be well identified. Bilateral distal salpingectomies and lysis of adhesions were performed. The patient tolerated this procedure well, was extubated in good condition, and was taken back to the recovery room.

Pathological examination confirmed the presence of immature chorionic villi of the left fallopian tube and immature chorionic villi and hemorrhage of the right fallopian tube, consistent with BTP. The patient was discharged home on postoperative day two and advised to follow up in our clinic in two weeks. However, the patient did not attend her scheduled follow-up appointment.

## Discussion

An increase in BTP cases has been observed mainly due to the use of ART. The specific factors favoring ectopic implantation with ART include transferring multiple embryos, using fresh versus cryo-thawed embryos, and transferring cleavage-stage versus blastocyst embryos. There is a 2.1-8.6% chance of EP with in-vitro fertilization in comparison to 2% with spontaneous conception [2].

Several hypotheses have been made to explain the cause of spontaneous BTP. The theories mentioned in the literature include damage to the fallopian tubes, which, in turn, could cause the retention of an embryo; the migration of trophoblastic cells from one tube to another; and the development of a second oocyte in an established pregnant woman [3,5]. Fishback proposed in 1939 that the diagnosis of BTP should entail the observation of two embryos and chorionic tissue in the fallopian tubes [6]. Fourteen years later, Norris altered this definition to only requiring chorionic villi in both tubes in which our patient met diagnostic criteria [7].

Morbidity and mortality are of the utmost concern regarding EP, and even more so when both fallopian tubes are involved, which increases the risk of rupture and hemorrhagic shock [8]. EP rupture has an overall incidence of 5-10% of all pregnancy-related deaths [2]. The signs and symptoms are crucial to diagnosing EP early and do not differ for a unilateral or bilateral EP [1]. The classic symptom triad consists of amenorrhea, vaginal bleeding, and abdominal pain [9]. These nonspecific symptoms present clinically similar to and are often misdiagnosed as other conditions such as appendicitis, urinary calculi, early pregnancy loss, and trauma [2].

The initial diagnostic tools for EP include ultrasound imaging and β-hCG monitoring. However, it can be argued that these methods are not the “gold standard.” US imaging is dependent on manual, learned skills and β-hCG levels do not correlate with the site of implantation. One review analyzed 42 cases of BTPs, 21 of which were spontaneous, and none were correctly diagnosed before surgery [1,10]. While the efficacy of US imaging is in question, the method of US does appear to make a difference. Three-dimensional transvaginal US combined with color Doppler has shown to be more accurate and sensitive in EP detection in comparison to transabdominal US. Despite the confirmation of an EP with US imaging and a β-hCG level >2,000 mIU/mL with no sign of an intrauterine pregnancy, the possibility of a BTP must be further investigated [2]. Other diagnostic tools have been investigated, such as monitoring white blood cell counts, specifically monocyte counts, platelet distribution width, activin-AB, plasma protein A, and endometrial sampling techniques. However, the literature available on these methods is quite limited [2].

MTX is the current standard for use in hemodynamically stable patients with EP. It functions as a folate antagonist to inhibit rapid cell division. MTX is prescribed in single, double, or multi-dose regimens depending on the patient’s β-hCG trends. It can be administered intramuscularly or injected directly into the fallopian tubes [2,10]. Rising β-hCG levels post-treatment are suggestive of treatment failure. The current literature estimates the resolution of unilateral EP with MTX to be between 70-95%, with lower success rates in patients with higher initial β-hCG levels [2]. At this time, there are no treatment guidelines or reports of successful treatment of BTP with MTX [1,10]. Management appears to be dependent on the stability of the patient, the extent of tubal damage, and the desire for future fertility [10].

While medical management is ideal for conservative treatment, surgery tends to be the most efficient mechanism for BTP diagnosis and treatment. Of the surgical treatments, salpingostomy is deemed the better choice for patients with a desire for future fertility. It consists of making an incision at the fallopian tube for removal of the EP, leaving the tube intact. Salpingectomy is the removal of part or all of the fallopian tube, which puts future fertility at decreased odds, but significantly reduces the chance for recurrent EP. Salpingectomy is generally advised for patients with EPs >5 cm, as well as for tubal damage and rupture. Pathologic findings of EP confirm the success of this procedure. However, patients undergoing salpingostomy should be followed with serial β-hCG measurements to rule out persistent trophoblastic tissue [2,5].

## Conclusions

A bilateral EP should not be crossed off the list of differential diagnoses despite the confirmation of a unilateral EP identified with diagnostic imaging. Our patient was medically managed based on the findings in the ultrasound report, which led to sub-therapeutic treatment and, ultimately, fallopian tube rupture. As there is no definitive algorithm or guidelines to follow for BTP, diagnosis and treatment are often achieved with laparoscopic intervention. While the preservation of future fertility is in the best interest, this cannot be attained without early recognition.
